# Preoperative Serum Sex Hormone-Binding Globulin Level Is an Independent Predictor of Biochemical Outcome After Radical Prostatectomy

**DOI:** 10.1097/MD.0000000000001185

**Published:** 2015-07-17

**Authors:** Jung Keun Lee, Seok-Soo Byun, Sang Eun Lee, Sung Kyu Hong

**Affiliations:** From the Department of Urology, Seoul National University of Hospital, Seoul (JKL); and Department of Urology, Seoul National University Bundang Hospital, Seongnam, Gyeonggi-do, Korea (S-SB, SEL, SKH).

## Abstract

To investigate the significance of preoperative serum sex hormone-binding globulin (SHBG) level regarding the postoperative biochemical outcome in patients who were followed up for relative longer periods after undergoing radical prostatectomy (RP).

Preoperative serum levels of testosterone (T), free T, and SHBG level were prospectively analyzed in 307 consecutive patients who underwent RP at our institution between January 2006 and July 2007. We analyzed potential associations of sex hormones with postoperative biochemical recurrence (BCR)-free survival via multivariate Cox proportional regression analysis.

Mean postoperative follow-up duration for 307 total patients was 72.1 ± 19.6 months. Kaplan–Meier curve demonstrated that BCR-free survival was significantly worse in patients with higher (≥ 40 ng/mL) SHBG level than others (*P* < 0.001). Serum T (*P* = 0.280) and free T (*P* = 0.606) levels showed no significant association with biochemical outcome. In multivariate analysis encompassing postoperative variables along with PSA, T, and free T, SHBG level (HR 1.825, 1.061–3.138; *P* = 0.030) was observed to be independently associated with BCR-free survival. Addition of SHBG level to the multivariate model for prediction of BCR-free survival resulted in increased accuracy (83.5% vs. 82.2%; *P* = 0.164).

Our study of patients who were followed up for relative longer periods after RP shows that preoperative serum SHBG level, but not T, is an independent predictor of postoperative BCR-free survival. According to our findings, SHBG measurement may be useful in the selection of candidates for adjuvant treatment following RP.

## INTRODUCTION

Prostate is considered an organ dependent on sex steroids, mainly testosterone (T).^1^ Accordingly, robust evidence has suggested that sex hormones have significant role in the etiology and pathogenesis of prostate cancer (PCa).^2,3^ For decades, biological dependence between PCa and T was assumed.^1–3^ However, such assumption has yet to be confirmed clearly via epidemiologic studies.^4,5^ Although several groups have investigated the prognostic significance of serum levels of various sex hormones, mainly T, regarding PCa, findings were inconsistent and inconclusive.^5–8^ Controversy continues on the actual value of T level as a prognosticator in PCa.^9,10^

Sex hormone-binding globulin (SHBG), which binds to the hormones and prevents their diffusion across cell membranes, is known to regulate the bioavailability of sex steroids.^11,12^ SHBG has also been widely applied in the assessment of bioavailable T level.^11^ Also, serum SHBG level has been reported to be a relatively stable parameter, not showing timely fluctuation as with serum T level.^11^ Meanwhile, it has been reported that binding capacity of SHBG for steroids may be altered in hormone-dependent cancers such as PCa.^13^ Moreover, SHBG level has been shown to be higher in PCa patients than in healthy controls.^11^ Previously, from analyzing men undergoing radical prostatectomy (RP) from 2006 to 2007, we reported that serum SHBG level was an independent predictive factor for extraprostatic extension of tumor in patients with clinically localized PCa, a finding also confirmed later in a similar study by others.^6,7,14^ In addition, SHBG has been reported to be associated with lymph node involvement in men undergoing RP.^15^ These published data would indicate that SHBG level may well have a role in the prediction of prognosis, at least in patients undergoing RP. Looking at the literature, not many have reported on the significance of SHBG level on the biochemical outcome after RP.^6,7^ Thus, we investigated the prognostic significance of preoperative serum SHBG level in patients undergoing RP by analyzing the follow-up data of patients who underwent RP, mostly from our previous study aforementioned.^14^

## METHODS

### Study Patients

With the approval of our institutional review board, we reviewed the records of consecutive patients who had serum total T, free T, and SHBG level measured preoperatively and underwent RP at our institution between January 2006 and July 2007. We excluded patients who had medical problem which could affect serum sex hormone level, such as thyroid disease, liver disease, uncontrolled diabetes mellitus, hyperprolactinemia, and hypoalbuminemia. Any patients who had history of other malignancies and/or undergone neoadjuvant/adjuvant treatments for prostate cancer were also excluded from analysis. With 19 more patients (mostly underwent RP in July 2007) newly added to 288 patients who were subjects of our previously reported study, a total of 307 patients were included in the present study.

### Hormones Assayed

Preoperatively, serum samples for hormone evaluation were obtained via venous tract at 7:30 and 10:00 am, centrifuged for 7 minutes, and stored immediately at −80 °C.^14^ T and free T levels were, respectively, measured by chemiluminescent microparticle immunoassay using Architect Testosterone Reagent kit (Abbott Laboratories, Chicago, IL) and radioimmunoassay using Coat-A-Count Free Testosterone kit (DPC, Los Angeles, CA). Serum SHBG levels were measured by an immunoradiometric assay with the use of the IRMA-count SHBG kit (DPC, Llanberis, UK). Each sample was assayed for each analyte in duplicate, the average of 2 values taken for analysis. Coefficient of variation was <8% for each assay.

### Analyzed Parameters and Follow-Up Protocol

From our prospectively managed RP database, data on various clinicopathologic factors including age, body mass index (BMI), prostate volume, PSA level, clinical stage, biopsy/pathologic Gleason score (GS), pathologic stage, and marginal status were obtained. TNM stage and GS were assigned according to the 2002 TNM classification and the criteria of the International Society of Urological Pathology, respectively.^16,17^ After RP, all patients were followed routinely at our outpatient clinic. PSA was checked every 3 months after discharge for the 1 year and 6 months thereafter. Postoperative biochemical recurrence (BCR) was defined as having PSA ≥0.2 ng/mL at least after 1 month following RP. Patients who had no evidence of BCR after RP were censored at the date of the last follow-up.

### Statistical Analysis

In this study, SPSS software, version 19.0 (IBM, Chicago, IL) was used for statistical analysis. First, potential associations between serum hormone level and various clinicopathologic parameters were analyzed via Student *t* test and *χ*^2^ test, while significance of correlations among hormone levels was analyzed with Spearman test. Second, multivariate logistic regression analyses were performed to identify significant preoperative predictors of adverse pathologic features (pathologic stage ≥T3 or higher and/or pathologic Gleason score ≥4 + 3). Third, Kaplan–Meier curve was used to analyze the effects of hormone levels on BCR-free survival. And, multivariate Cox regression analysis was used to assess the correlations between clinicopathologic variables and BCR-free survival. Also, receiver operator characteristics (ROC) curves were obtained to demonstrate predictive performances of multivariate models. Area under curves (AUCs) were compared via Mantel–Haenszel test. All *P* values <0.05 were considered statistically significant.

## RESULTS

Table 1 shows clinicopathologic characteristics of 307 total patients included in this study. As shown in Table 1, patients with higher SHBG level were significantly older and also had lower BMI, higher T level, and higher PSA level than counterparts with lower SHBG level (all *P* values <0.05). Also, men with higher SHBG level were observed to have higher biopsy Gleason score, higher pathologic stage, and higher pathologic GS (all *P* values <0.05). There was a positive association of SHBG with T (*ρ* = 0.557, *P* < 0.001), but no significant association with free T (*ρ* = 0.092, *P* = 0.110).

**TABLE 1:**
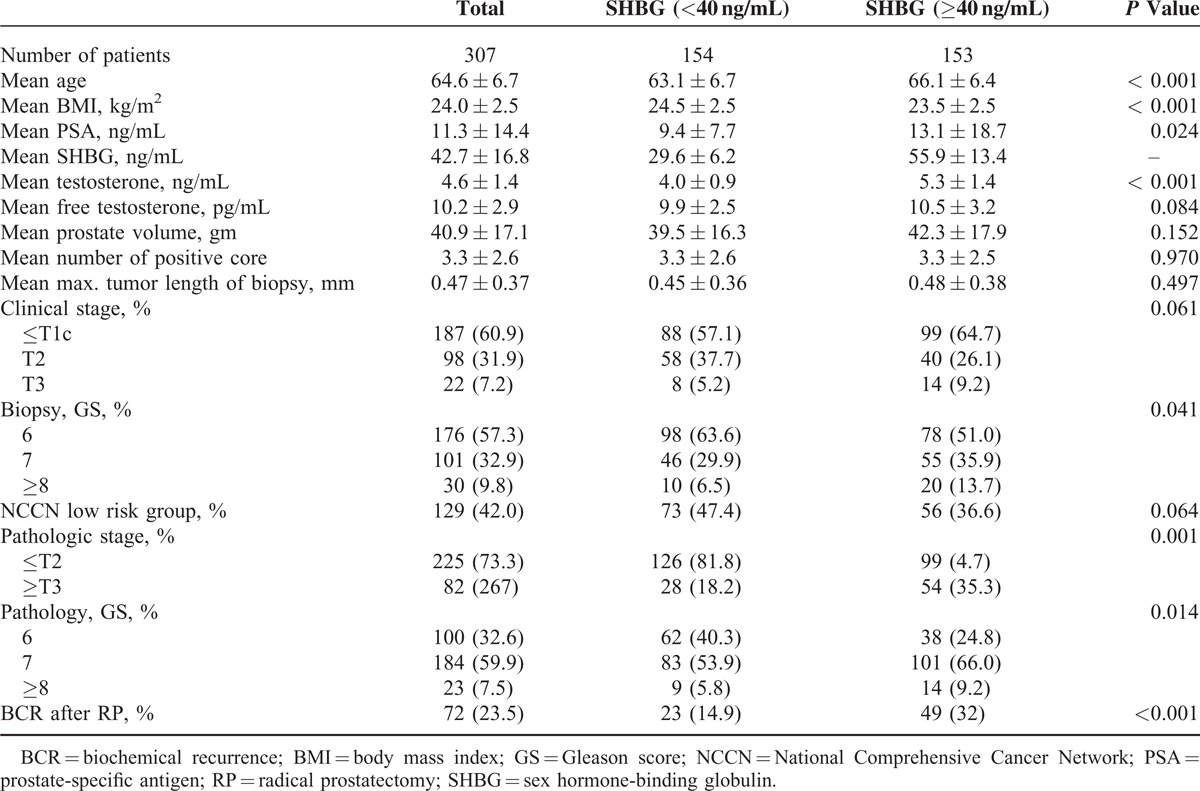
Clnicopathological Characteristics of 307 Patients

In multivariate logistic regression analysis, higher SHBG was found to be an independent predictor of adverse pathologic features (OR 2.503, 1.207–5.190; *P* = 0.014) along with PSA (OR 1.097, 1.047–1.150; *P* < 0.001), prostate volume (OR 0.964, 0.941–0.987; *P* = 0.002), and biopsy GS (OR 9.674, 2.079–18.425; *P* = 0.001). However, T level (OR 1.418, 0.678–2.967; *P* = 0.354) and free T level (OR 0.916, 0.461–1.819; *P* = 0.802) demonstrated no such significant correlations.

**FIGURE 1:**
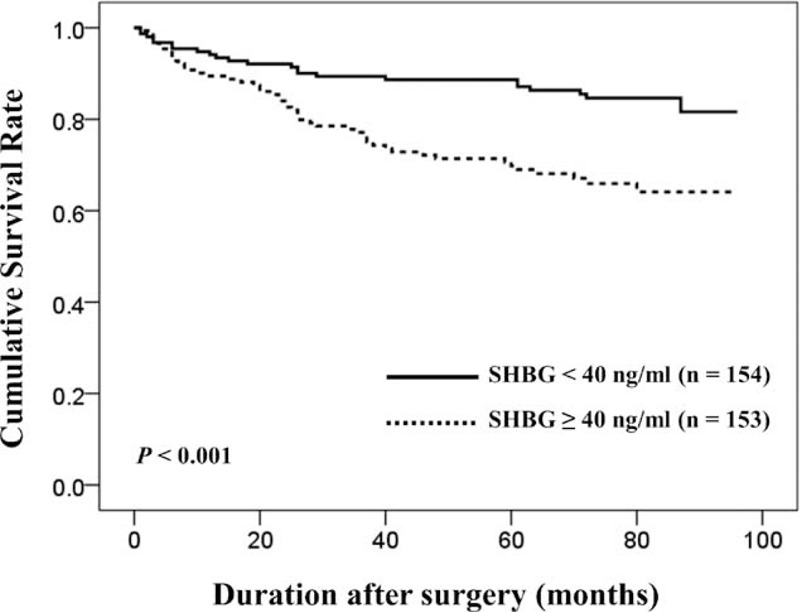
Kaplan–Meier estimates of biochemical recurrence-free survival after radical prostatectomy in the low (<40 ng/mL) and high (≥40 ng/mL) preoperative serum SHBG groups among the total subjects. The biochemical recurrence-free survival rate was lower in high SHBG group (*P* < 0.001). SBHG = sex hormone-binding globulin.

**FIGURE 2:**
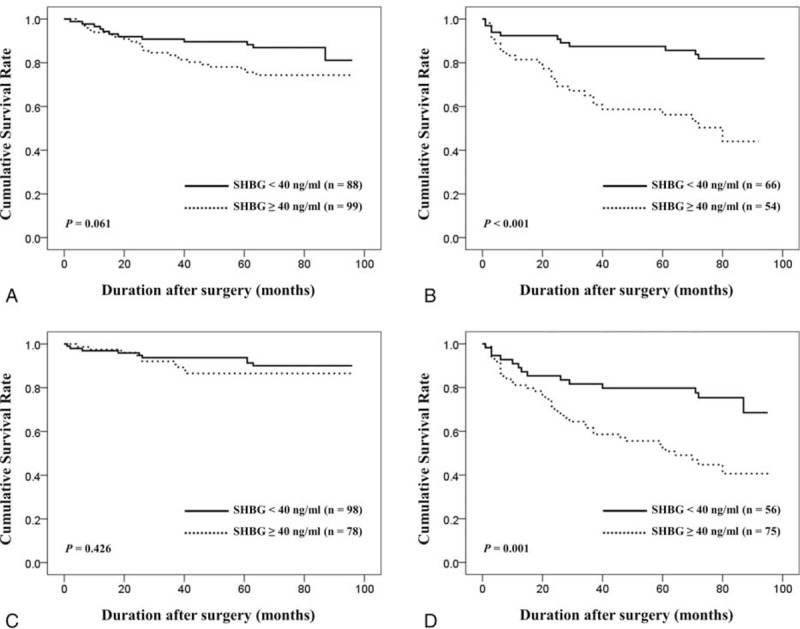
Kaplan–Meier estimates of biochemical recurrence-free survival after radical prostatectomy in the low (<40 ng/mL) and high (≥40 ng/mL) preoperative serum SHBG groups among the 4 different subgroups: clinical stage ≤T1c (A), clinical stage ≥T2a (B), biopsy Gleason score ≤6 (C), and biopsy Gleason score ≥7 (D). SHBG = sex hormone-binding globulin.

Mean and median (IQR) postoperative follow-up duration for 307 total patients included in our study were 72.1 ± 19.6 months and 77.0 (67.0–84.0) months, respectively. During postoperative follow-up periods, BCR occurred in 23.5% of all subjects. Among the total subjects, the BCR-free survival at 36 and 60 months was 83.3% and 79.3%, respectively. The Kaplan–Meier method demonstrated that patients within higher SHBG level had worse BCR-free survival than others (*P* < 0.001) (Figure 1). However, T (*P* = 0.280) and free T (*P* = 0.606) were not associated with BCR-free survival. Additionally, we analyzed the significance of SHBG level regarding BCR-free survival according to clinical stage (≤T1c vs. ≥T2a) and biopsy GS (≤6 vs. ≥7) through subgroup analyses. As shown in Figure 2, patients with higher SHBG level also had significantly worse biochemical outcome among the subgroup of patients with clinical stage ≥T2a (*P* < 0.001) and those with biopsy GS ≥7 (*P* = 0.001). Meanwhile, such phenomenon was not observed among the subgroup of patients with clinical stage ≤T1c (*P* = 0.061) or those with biopsy GS ≤6 (*P* = 0.426).

In univariate analyses, age, PSA level, biopsy GS, pathologic GS, pathologic stage, positive surgical margin, and SHBG level were associated with BCR-free survival (all *P* values < 0.05). In multivariate Cox proportional regression analysis encompassing preoperative variables only (age, PSA, T level, free T level, BMI, prostate volume, clinical stage, and biopsy GS), SHBG level was observed to be significantly associated with worse postoperative BCR-free survival (HR 2.005, 1.121–3.587; *P* = 0.019) (Table 2a). Along with SHBG level, PSA level and biopsy GS were also found to be significantly associated with BCR-free survival in the multivariate analysis. Meanwhile, in multivariate Cox proportional regression analysis encompassing postoperative variables along with PSA, T, free T, and SHBG, serum SHBG level (HR 1.825, 1.061–3.138; *P* = 0.030) was also found to be an independent predictor for BCR-free survival along with pathologic stage and pathologic GS (Table 2b).

**TABLE 2:**
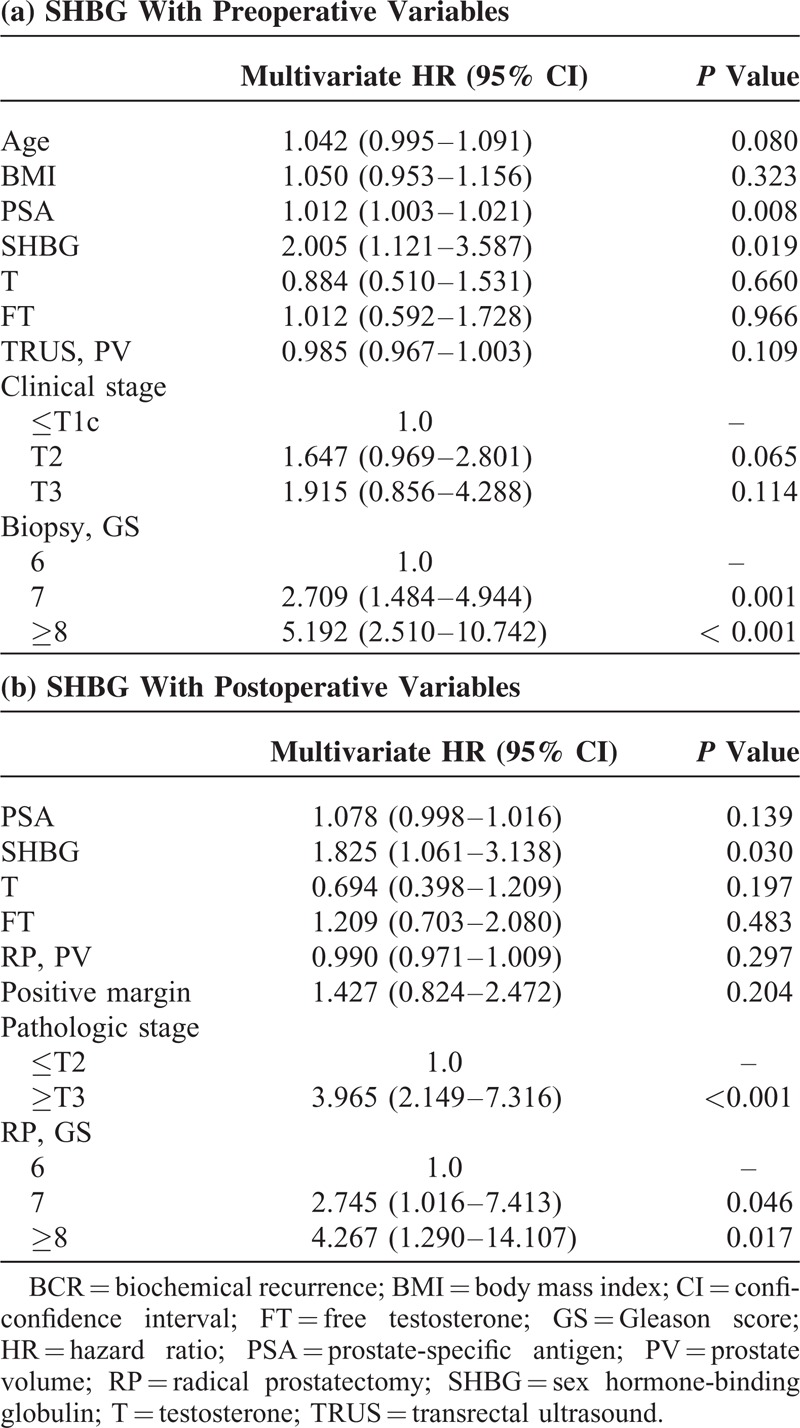
Cox-Proportional Regression Analysis to Identify Significant Predictors for Biochemical Recurrence-Free Survival After Radical Prostatectomy

We constructed a base multivariate logistic regression model (MLRM) for the prediction of postoperative BCR encompassing the following preoperative variables only: age, BMI, T, free T, PSA, TRUS volume, clinical stage, and biopsy GS. Addition of SHBG level to the base model resulted in increased predictive accuracy of the model (80.3% vs. 78.5%; *P* = 0.473). Also, we constructed another MLRM using the following variables: PSA, T, free T, RP specimen weight, pathologic stage, pathologic GS, and marginal status. Similar to the first model, addition of SHBG level to this second model contributed to increased predictive accuracy (83.9% vs. 82.2%; *P* = 0.164).

## DISCUSSION

In this follow-up study of our previous investigation on the relationship of serum SHBG level with known prognostic factors for PCa, we investigated the significance of preoperative SHBG level regarding biochemical outcome following RP.^14^ It should be noted that mean follow-up duration of our subjects was more than 70 months. In the present study, we observed that preoperative serum SHBG level, adjusted for levels of T and free T, was an independent predictor of postoperative BCR in men who underwent RP. Such finding was observed regardless of whether multivariate model included pre- or postoperative variables. In our study, T and free T levels were not observed to be associated with biochemical outcome following RP. If corroborated by others, serum SHBG level would prove helpful in the identification of patients at high risk of progression following RP.

Compared with T, data on the association of PCa and SHBG are relatively scarce. Raivio et al observed that SHBG level was not significantly different between men with benign prostatic hyperplasia (BPH) and PCa.^18^ Also others reported similar findings on the association between SHBG level and presence of PCa.^2,3,19^ However, Grasso et al observed significantly higher SHBG level in PCa patients than those with BPH or healthy controls.^13^ Meanwhile, they could not confirm any association between SHBG and extent or stage of PCa and suggested that plasma-binding globulin is heterogenous. By comparing PCa patients with and without metastasis, Haapiainen et al reported that patients with metastatic disease showed significantly lower pretreatment T/SHBG ratio than nonmetastatic counterparts, whereas they observed no significant difference in T levels.^20^ From their findings, they mentioned that predictive value of T/SHBG ratio as a prognostic factor was higher than that of T level. It should be noted that our multivariate models also included both serum T and SHBG levels. As aforementioned, our group previously reported that preoperative SHBG level along with serum PSA level, biopsy Gleason score, and clinical stage was an independent predictor of extraprostatic extension of tumor in patients undergoing RP for clinically localized PCa.^14^ Salonia et al performed a similar study by measuring SHBG levels in 629 European men undergoing RP and also found that SHBG level was significantly associated with extracapsular extension, confirming our finding.^21^ The same group also reported that preoperative SHBG level was a significant multivariate predictor of lymph node invasion in men who underwent RP.^15^

Many groups have investigated the potential association of sex hormones, mainly T level, and biochemical outcome in men undergoing RP.^6,7,20,22^ Although several relevant studies demonstrated that low preoperative T level was associated with higher risk of postoperative BCR, others have reported to the contrary.^23,24^ In our study, T and free T were not observed to be significant predictor of biochemical outcome following RP. Looking at such controversy, it can be suggested that implementation of more reliable surrogate for assessing systemic androgenecity, such as SHBG level, might be appropriate for the prediction of outcomes among PCa patients undergoing treatment. As aforementioned, there are only sparse data currently available on the prognostic significance of SHBG. Analyzing 285 men undergoing RP, Waldert et al observed that preoperative serum SHBG level was independently associated with BCR after RP.^6^ They reported that for each 10 U increase in SHBG the risk of BCR increased by 12%. Similar to our findings, T and free T were not associated with BCR in Waldert et al's series. Salonia et al reported from analyzing 793 European men undergoing RP that preoperative serum SHBG level along with T, 17β estradiol, high biopsy Gleason score achieved independent predictor status for early BCR (defined as when the first PSA value ≥0.1 ng/mL occurred within 24 months after RP).^7^ Despite the differences in analytic methodology, including variables analyzed and BCR definition applied, these previous reports corroborate our findings. Unlike the 2 aforementioned studies, we included both BMI and prostate volume in multivariable models to analyze the potential role of SHBG in the prediction of BCR since both variables can be considered hormone-dependent and have been reported as potential prognostic factor in various RP series.^25,26^ Furthermore, it should be noted that serum SHBG level was shown to be an independent predictor of postoperative biochemical outcome regardless of T level and whether preoperative or pathologic variables were incorporated into multivariable model in our study. In our subgroup analyses, serum SHBG level was not observed to be a significant predictor of biochemical outcome among the subgroups of patients with relatively less aggressive disease (≤T1c or biopsy GS ≤6) in univariate analyses. Such phenomenon may well be largely attributed to more favorable outcome (lower recurrence rate) of low-risk PCa. Meanwhile, findings may have been different with a larger cohort.

As for the possible explanations for observed association between SHBG and biochemical outcome in men undergoing RP, currently no definitive conclusion can be offered. Hryb et al showed that SHBG is produced by human PCa cells as well as cultured human prostate epithelial and stromal cells.^27^ Such observation would imply that SHBG may be locally regulated and produced, potentially having direct influence on carcinogenesis and/or progression of PCa independent of T. It can also be hypothesized that SHBG may additionally have indirect effects on carcinogenesis and/or progression of PCa. Specific binding sites for SHBG on prostate cell membranes have been described.^27^ Also, it has been reported that SHBG can stimulate intraprostatic production of cyclic adenosine monophosphate (cAMP), and that androgen receptors in prostate can be activated by growth factors or cAMP.^28^ Thus, it can be hypothesized that SHBG increases the responsiveness of prostate epithelium to T, possibly leading up to carcinogenesis and/or cancer progression. Meanwhile, data from genetic polymorphisms involved in metabolic pathways of sex hormones suggest that interindividual variations in genes associated with sex hormones could be related with PCa risk.^29^ Also, previous studies have shown that single-nucleotide polymorphisms (SNP) in *SHBG* gene were associated with serum T level as well as serum SHBG level.^30^ Accordingly, it can be hypothesized that polymorphisms in *SHBG* locus may be related to PCa aggressiveness. Although a previous study found that SNPs in *SHBG* locus which were associated with plasma T and SHBG level did not match SNPs associated with PCa aggressiveness, we believe that our findings warrant further investigations on the role of SHBG in pathogenesis of PCa.^29^

Our study is not devoid of limitations. Potential limitations include relatively small size of patient cohort compared with other RP series. Also as only RP cohort was analyzed, our findings may not be applicable to patients undergoing other forms of treatment. In addition, postoperative changes in serum levels of SHBG and other androgens were not analyzed in this study. Meanwhile, as others recently reported that SHBG, associated with poor clinical feature of PCa, is an important factor in stemness induction of cells by dihydroT in vitro, additional analyses on the expressions of various stemness related factors, such as Oct3/4 and Nanog, using tissues samples from RP specimens should be performed in the future.^31^ Overall, we believe that our findings warrant further investigation via a larger cohort of patients.

## CONCLUSIONS

Findings from the present study suggest that preoperative serum SHBG level is an independent predictor of biochemical outcome after RP. Conversely, T and free T levels may not be useful predictors of outcome following RP. According to our findings, preoperative SHBG measurement may be useful in the selection of candidates for adjuvant treatment following RP. Further investigations preferably via a larger cohort are needed to confirm these results.
